# Diffusivities and Atomic Mobilities in bcc Ti-Mo-Zr Alloys

**DOI:** 10.3390/ma11101909

**Published:** 2018-10-08

**Authors:** Weimin Bai, Guanglong Xu, Mingyue Tan, Zhijie Yang, Lijun Zeng, Di Wu, Libin Liu, Ligang Zhang

**Affiliations:** 1 School of Material Science and Engineering, Central South University, Changsha 410083, China; morrisbai@csu.edu.cn (W.B.); tmy_luna@163.com (M.T.); yangzhijiecsu@163.com (Z.Y.); ljzeng.mse@csu.edu.cn (L.Z.); wudi080133@163.com (D.W.); 2 Tech Institute for Advanced Materials & School of Materials Science and Engineering, Nanjing Tech University, Nanjing 211800, China; guanglongxu@njtech.edu.cn

**Keywords:** bcc Ti-Mo-Zr alloys, Inter-diffusion coefficient, Impurity coefficient, Atomic mobility, CALPHAD modeling

## Abstract

β-type (with bcc structure) titanium alloys have been widely used as artificial implants in the medical field due to their favorable properties. Among them, Ti-Mo alloy attracted numerous interests as metallic biomaterials. Understanding of kinetic characteristics of Ti alloys is critical to understand and manipulate the phase transformation and microstructure evolution during homogenization and precipitation. In this work, diffusion couple technique was employed to investigate the diffusion behaviors in bcc Ti-Mo-Zr alloys. The diffusion couples were prepared and annealed at 1373 K for 72 h and 1473 K for 48 h, respectively. The composition-distance profiles were obtained via electron probe micro-analysis (EPMA). The chemical diffusion coefficients and impurity diffusion coefficients were extracted via the Whittle-Green method and Hall method. The obtained diffusion coefficients were assessed to develop a self-consistent atomic mobility database of bcc phase in Ti-Mo-Zr system. The calculated diffusion coefficients were compared with the experimental results. They showed good agreement. Simulations were implemented by Dictra Module in Thermo-Calc software. The predicted composition-distance profiles, inter-diffusion flux, and diffusion paths are consistent with experimental data, confirming the accuracy of the database.

## 1. Introduction

Due to their biocompatibility, low modulus, high specific strength, and high corrosion resistance, β-type (with bcc structure) and near β titanium alloys have been widely used as surgery implants [1,2,3,4]. Previous studies have verified that Nb, Zr, Mo, Hf, and Ta are effective alloying elements leading to low elastic modulus, nontoxic, and non-allergic β-Ti alloys [4,5,6]. Moreover, Zr can increase hardenability and corrosion resistance for the alloy [7,8]. Mo is a strong β-phase stabilizing element for titanium alloys and the Ti-Mo based alloys exhibit adequate mechanical compatibility and good cyto-compatibility [9,10,11,12]. Therefore, the Ti-Mo-Zr system, which showed good performances and magnificent prospects in biomaterial application, has been intensively studied [3,6,10,11,12,13]. Several alloys related to the Ti-Mo-Zr system have been investigated recently, for example, Ti-12Mo-6Zr-2Fe [14], Ti-15Mo-5Zr-3Al [15], and Ti-8Mo-4Nb-2Zr [6].

Inter-diffusion in alloys plays an important role in materials processing. Understanding diffusion kinetics of Ti alloys is critical to manipulate phase transformation and microstructure evolution during homogenization and precipitation [16,17,18]. The reliable inter-diffusion coefficients are the essential input parameters in various quantitative modeling of phase transformation [17,19,20]. It is also valuable to determine thermodynamic stability under long-term service conditions.

Inspired by the CALPHAD (CALculation of PHAse Diagram) method, Andersson and Ågren [21] developed a phenomenological kinetic technique which present the atomic mobilities of individual species using Redlick-Kister polynomials. This technique assesses the discrete diffusion coefficients that were extracted experimentally and then employs a set of parameters to describe composition dependent mobilities and diffusivities. Combined with thermodynamic information, diffusion simulation can be implemented in DICTRA module embedded in Thermo_Calc software [22,23].

Various of measurements of self- and impurity diffusion coefficients in bcc Ti, Zr, and Mo have been conducted [24] as well as inter-diffusion coefficients in bcc Ti-Mo [25,26,27], Ti-Zr [28,29,30,31] and Mo-Zr alloys [32]. Based these diffusion coefficients, Liu et al. [33,34] assessed the atomic mobilities in bcc Ti-Mo, Ti-Zr, and Mo-Zr alloys with the CALPHAD method. Simulations of concentration profiles and Kirkendall movement using these mobility parameters show good agreement with the experimental results from literatures. However, the ternary diffusion coefficients and mobilities in bcc Ti-Mo-Zr alloys is still open to be evaluated and barely reported. The purpose of this work is to study the diffusion behavior of Mo and Zr in β-Ti alloys and establish a CALPHAD-type atomic mobility database of bcc phase in Ti-Mo-Zr system.

## 2. Model 

### 2.1. Extraction of Inter-Diffusion Coefficients and Impurity Diffusion Coefficients

According to Kirkaldy [35], in one dimension, the inter-diffusion flux of component *i* with concentration *c_i_* in a ternary system can be expressed as:

(1) $${\tilde{J}}_{i}=\frac{1}{2t}\int_{c_{i}^{-}}^{c_{i}}\left(z_{i}-z_{0}\right)dc_{i}=-\sum_{j=1}^{2}{\tilde{D}}_{ij}^{k}\frac{\partial c_{j}}{\partial z},$$

where ${\tilde{D}}_{ij}^{k}$ are the inter-diffusion coefficients;$c_{i}$, $c_{j}$ are the concentration of species i and j; $c_{i}^{-}$ is the concentration of i at the left end of the diffusion couple; z is the distance; $z_{0}$ is the Matano plane for the diffusion couple; and, t is the diffusion time.

In a ternary system, it involves four independent diffusion coefficients, ${\tilde{D}}_{11}^{3}$, ${\tilde{D}}_{22}^{3}$, ${\tilde{D}}_{12}^{3}$ and ${\tilde{D}}_{21}^{3}$. However only two composition-distance profiles for independent elements 1 and 2 can be derived from one diffusion couple. Hence, two diffusion couples result in two diffusion paths which intersect at one point, are needed to obtain four profiles. Then, the diffusion coefficients at this point can be solved mathematically. The old-fashioned Boltzmann-Matano had to position the Matano planes of four profiles, which is time-consuming and might lead to unnecessary inaccuracy. To avoid this drawback, Whittle and Green [36] introduced a normalized concentration variable:

(2) $$Y_{i}=\frac{c_{i}-c_{i}^{-}}{c_{i}^{+}-c_{i}^{-}},$$

where $c_{i}^{-}$ and $c_{i}^{+}$ are concentrations of two elements in the left and right end of the diffusion couple.

With the variable *Y*, the inter-diffusion flux of element *i* is no longer referred to a fixed laboratory coordinate and can be transformed into:

(3) $${\tilde{J}}_{i}=\frac{\left(c_{i}^{+}-c_{i}^{-}\right)}{2t}\left[\left(1-Y_{i}\right)\int_{-\infty }^{z}Y_{i}dz+Y_{i}\int_{-\infty }^{z}\left(1-Y_{i}\right)dz\right].$$

Reformulating the right-hand side of Equations (1) and (3) lead to:

(4) $$\frac{1}{2t}\left(\frac{dz}{dY_{i}}\right)\left[\left(1-Y_{i}\right)\int_{-\infty }^{z}Y_{i}dz+Y_{i}\int_{-\infty }^{z}\left(1-Y_{i}\right)dz\right]={\tilde{D}}_{ii}^{k}+{\tilde{D}}_{ij}^{k}\frac{dc_{j}}{dc_{i}}\left(i=1,2\right).$$

By solving a set of four equations from one pair of diffusion couples, the diffusion coefficients ${\tilde{D}}_{MoMo}^{Ti}$, ${\tilde{D}}_{ZrZr}^{Ti}$, ${\tilde{D}}_{MoZr}^{Ti}$ and ${\tilde{D}}_{ZrMo}^{Ti}$ at the intersecting composition can be extracted. Note that the molar volume was taken to be constant for the lack of reliable data on the composition-dependent molar volume in bcc Ti-Mo-Zr alloys. Errors that are introduced by this approximate treatment are believed to be within the accuracy of the results obtained via the Whittle-Green method [36].

By applying a generalized Hall method [37,38], the impurity diffusion coefficients of Zr in Ti-Mo alloys and Mo in Ti-Zr at the terminal compositions of the diffusion couples can be obtained. The profiles were transformed to a plot of *μ vs λ*, in which erfμ=2Y−1 and $\lambda =z/\sqrt{t}$. By fitting the plot with a linear equation μ=hλ+k, the $h_{1}$, $k_{1}$ for the left terminal, and $h_{2}$, $k_{2}$ for the right terminal of the diffusion couples can be obtained and the impurity diffusion coefficients can be derived via the following formulas:

(5) $$\tilde{D}\left(z^{\prime }\right)=\frac{1}{4h_{1}^{2}}\left[1+\frac{2k_{1}}{\sqrt{\pi }}\exp\left(\mu^{2}\right)\times Y\left(z^{\prime }\right)\right],$$

(6) $$\tilde{D}\left(z^{\prime }\right)=\frac{1}{4h_{2}^{2}}\left[1-\frac{2k_{2}}{\sqrt{\pi }}\exp\left(\mu^{2}\right)\times \left[1-Y\left(z^{\prime }\right)\right]\right],$$

where $c_{i}$ represents the content of i in binary alloys and $c_{0}$ is the content of i at the other end of the diffusion couple.

### 2.2. Atomic Mobility and Diffusivity

As initiated by Andersson and Ågren [21] and later modified by Jönsson [39], the atomic mobility $M_{i}$ of species i can be expressed as:

(7) $$M_{i}=M_{i}^{0}\exp\left(\frac{-Q_{i}}{RT}\right)\frac{1}{RT}\times^{mg}\Omega =\exp\left(\frac{RT\ln M_{i}^{0}-Q_{i}}{RT}\right)\frac{1}{RT}\times^{mg}\Omega ,$$

where *R* is the gas constant; *T* is the temperature; $M_{i}^{0}$ is the frequency factor; $Q_{i}$ is the activation energy; and, ${}^{mg}\Omega$ is the ferromagnetic contribution. Since there is no ferromagnetic transition reported in Ti-Mo-Zr alloys, ${}^{mg}\Omega$ can be taken as 1. Provided $\Delta G_{i}^{\phi }=RT\ln M_{i}^{0}-Q_{i}$, Equation (7) can be written as [21]:

(8) $$M_{i}=\exp\left(\frac{\Delta G_{i}^{\phi }}{RT}\right)\frac{1}{RT},$$

the parameter $\Delta G_{i}^{\phi }$ is composition dependent and is expressed in the Redlich-Kister polynomial form as:

(9) $$\begin{array}{ll} \Delta G_{i}^{\phi } & =\sum_{p}x_{p}\Delta G_{i}^{p}+\sum_{p}\sum_{q>p}x_{p}x_{q}\left[\sum_{r=0,1,2,\ldots }\Delta^{\left(r\right)}G_{i}^{p,q}{\left(x_{p}-x_{q}\right)}^{r}\right]\\ & +\sum_{p}\sum_{q>p}\sum_{v>q}x_{p}x_{q}x_{v}\left\{\sum_{s=p,q,v}\left[x_{s}+\left(1-x_{p}-x_{q}-x_{v}\right)/3\right]\Delta^{\left(s\right)}G_{i}^{p,q,v}\right\} \end{array}$$

where ϕ denotes the solid solution phase; $x_{p}$ is the mole fraction of species p; $\Delta G_{i}^{p}$ is the value $\Delta G_{i}$ of species i in pure species p; and, $\Delta^{\left(r\right)}G_{i}^{p,q}$ and $\Delta^{\left(s\right)}G_{i}^{p,q,v}$ are the binary and ternary interaction parameters.

Assuming the mono-vacancy exchange is the dominant diffusion mechanism, and neglecting correlation factors, the tracer diffusion coefficients $D_{i}^{*}$ of species *i* is directly related to the mobility *M_i_* by means of the Einstein relation:

(10) $$D_{i}^{*}=RTM_{i},$$

For a substitutional solution phase, the inter-diffusion coefficients in terms of the volume-fixed reference frame is given by the following general expression:

(11) $${\tilde{D}}_{ij}^{n}=\sum_{k}^{n}\left(\delta_{ki}-x_{i}\right)x_{k}M_{k}\left(\frac{\partial \mu_{k}}{\partial x_{j}}-\frac{\partial \mu_{k}}{\partial x_{n}}\right),$$

where $\delta_{ki}$ is the Kronecker delta ($\delta_{ki}=1$ when *i=k*, and 0 otherwise).

Then, the inter-diffusion flux of species *i*, ${\tilde{J}}_{i}$ can be calculated via Equation (1), and the concentration evolution of component *i* in ternary systems can be solved by the mass conservation law:

(12) $$\frac{\partial c_{i}}{\partial t}+\frac{\partial {\tilde{J}}_{i}}{\partial z}=0.$$

What calls for special attention in the formula derivation is that the transformation of concentration of species *i*, $c_{i}$, and the mole fraction $x_{i}$:

(13) $$c_{i}=\frac{x_{i}}{V_{m}},$$

where *V_m_* is the molar volume of a phase that was taken to be constant in this work.

## 3. Experiment

**Figure 1:**
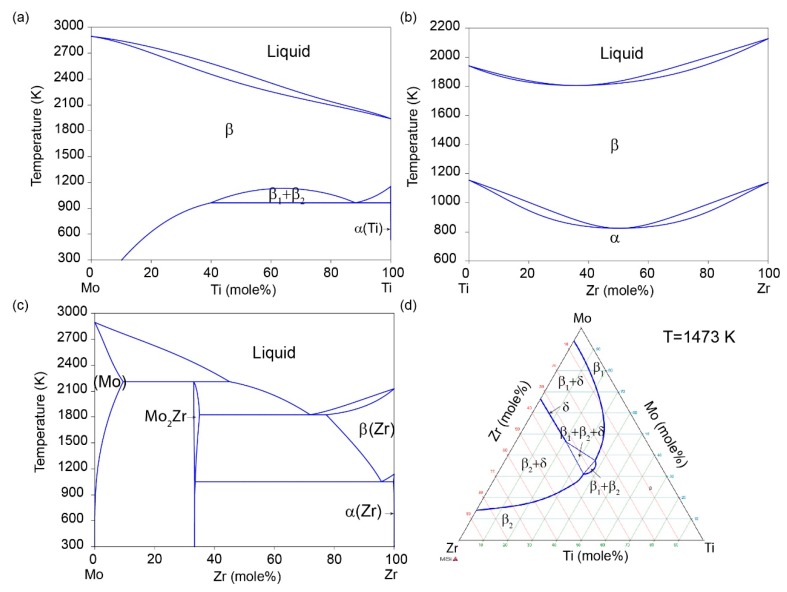
Phase diagram of (**a**) Ti-Mo [40], (**b**) Ti-Zr [41], (**c**) Zr-Mo [42], and (**d**) Ti-Mo-Zr [43] systems.

Pure Ti, binary Ti-Mo and Ti-Zr alloys, and ternary Ti-Mo-Zr alloys were prepared from pure Ti (99.99 wt %), Mo (99.99 wt %), and Zr (99.99 wt %) by arc melting in electric arc furnace under an argon atmosphere. All of the alloys compositions were designed in the bcc phase region according the binary phase diagram of Ti-Mo [40], Ti-Zr [41], and Zr-Mo [42] systems and ternary phase diagram of Ti-Mo-Zr system at 1473 K [43] (shown in Figure 1). The compositions of all alloys were listed in Table 1. The ingots were re-melted for six times to attain homogeneity and then annealed at 1473 K for 24 h to obtain microstructure with large grain size above millimeters, such that the effect of grain boundary diffusion can be ignored.

The annealed ingots were cut into blocks with a size of 10 × 10 × 5 mm using wire-electrode cutting. After one surface of the blocks polished to mirror-like quality, the well-contacted diffusion couples were assembled with appropriate pairs of blocks in Table 1 under vacuum at 1173 K for 4 h. The diffusion couples were sealed into quartz capsules that were filled with pure argon. The M1-M8 diffusion couples were annealed at 1373 K for 72 h and N1-N8 diffusion couples were annealed 1473 K for 48 h, followed by quenching in ice water.

After the annealing process, the diffusion couples were then cut into halves parallel to the ends using wire-electrode cutting, and then mounted, ground, and polished by standard metallographic techniques. The composition-distance profiles of 16 diffusion couples were determined using electron probe micro-analysis (EPMA, JEOL, JXA-8230, Tokyo, Japan, 15 kV, 20 nA) with 15 kV voltage, 20 nA current, and a 40° take-off angle. The accuracy of the EPMA test is >98% (mass percent).

## 4. Results and Discussions

### 4.1. Inter-Diffusion and Impurity Diffusion Coefficients At 1373 K and 1473 K

**Figure 2:**
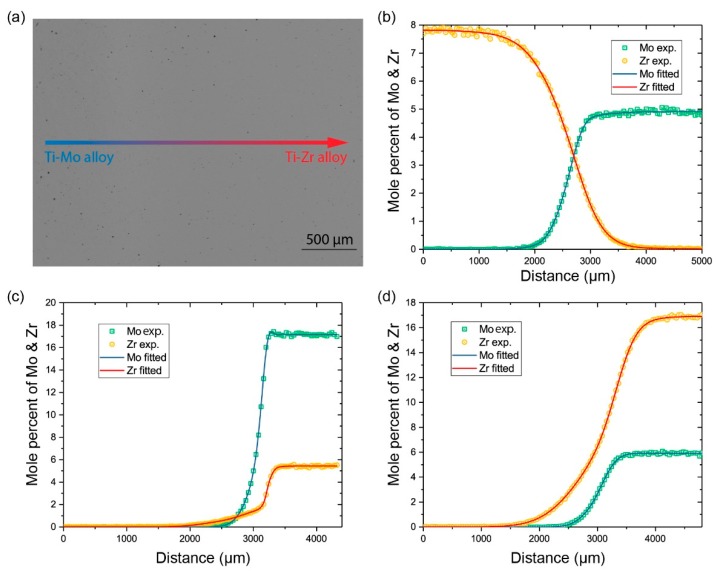
(**a**) Microstructure (BSE) of the diffusion couple M1 annealed at 1373 K for 72 h and the robust error function expansion (ERFEX) representation of the composition-distance profiles of the different couples annealed in 1373 K for 72 h: (**b**) for M2; (**c**) for M6; (**d**) for M8.

All of the diffusion couples show single bcc phase microstructure. The SEM backscattered electron image (BSE) of diffusion zone of the diffusion couple M1 is taken as an example and is presented in Figure 2a. There is no Kirkendal void existing in the diffusion zone and no microstructure is evident, revealing that the alloys were annealed in single bcc phase. After being annealed at high temperature and quenched, the martensite transformation may present in the samples. However, because martensite transformation is a diffusionless phase transformation, the composition profile will not change. We can still extract the diffusion coefficients from the composition profile that was obtained in this sample.

The composition-distance profiles that were determined from couple M2 is shown in Figure 2b. It is worth mentioning that, to avoid the errors from fitting or smoothing, a robust error function expansion (ERFEX) was put forward to represent the experimental profiles in an accurate analytical form [37,44]:

(14) $$X\left(r\right)=\sum_{i}\left[a_{i}\mathrm{erf}\left(b_{i}z+c_{i}\right)+d_{i}\right],$$

where *X*(*r*) is the effective alloying element content at location *z*; *a*, *b*, *c*, and *d* are the fitting parameters.

It could be evidenced by practice that the profiles smoothed using ERFEX method exhibits higher accuracy than using traditional method, such as moving average smoothing, Savitzky-Golay smoothing method, and Piecewise Cubic Hermite Interpolating Polynomial (PCHIP) interpolation, and it can present the details of the composition profiles. For example, the Mo pill-up at the right end of couple M6 (shown in Figure 2c). The ERFEX method also has its advantages in fitting asymmetrical curves when the diffusivities of the elements in one end of the diffusion couple are much larger than that of the other end. The couple M6 is compared with M8, for instance. The left-hand side of M6 is pure titanium and the other end has a high Mo concentration. The slop of profile M6 is steep at the right end of diffusion (Figure 2c). It is because the diffusivity of Mo in Ti alloys is much lower than that of Ti. In comparison, it is appropriately smooth and it shows the conventional S-shape (Figure 2d) in M8. The steep slope will introduce a larger error when calculating the diffusion coefficients at the concentration on it. That means that the accuracy of the calculated diffusion coefficients at the component close to Ti-Mo boundary will be lower.

**Figure 3:**
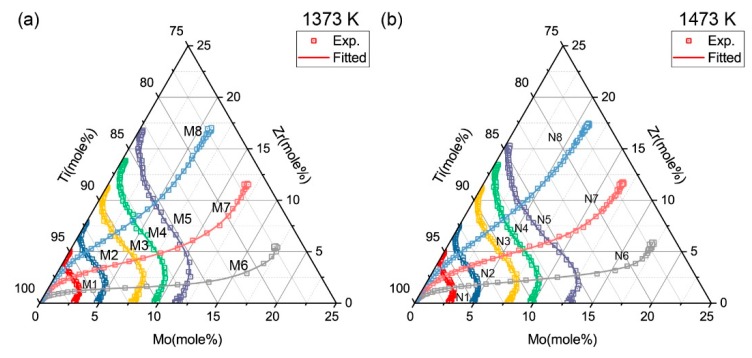
Diffusion paths of diffusion couples determined using electron probe micro-analysis (EPMA) after annealing process.

The diffusion paths of all diffusion couples after annealing process are presented in Figure 3. According to the Whittle-Green method, the inter-diffusion coefficients at the intersecting compositions can be extracted. The impurity diffusion of Zr in Ti-Mo alloys and Mo in Ti-Zr alloys can be obtained via the Hall method at the ends of couple M1-M5 and N1-N5.

**Figure 4:**
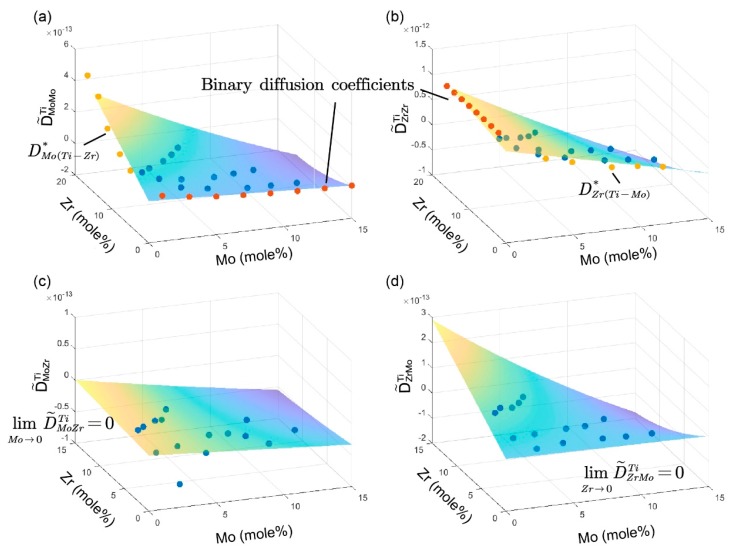
Three-dimensional (3D) plot of the ternary inter-diffusion coefficients (**a**) ${\tilde{D}}_{MoMo}^{Ti}$, (**b**) ${\tilde{D}}_{ZrZr}^{Ti}$, (**c**) ${\tilde{D}}_{MoZr}^{Ti}$ and (**d**) ${\tilde{D}}_{ZrMo}^{Ti}$ in the bcc Ti-Mo-Zr alloys at 1373 K, together with the impurity diffusion coefficients $D_{Mo\left(Ti-Zr\right)}^{*}$ and $D_{Zr\left(Ti-Mo\right)}^{*}$, and binary diffusion coefficients obtained from the literature [33].

The determined inter-diffusion coefficients are summarized in Table 2 and graphically presented with three-dimensional plots in Figure 4. The standard deviations were determined from four independent calculations upon two independent measurements. All of the results can satisfactorily fulfill the thermodynamic constrains derived by Kirkaldy [45]:

(15) $${\tilde{D}}_{ZrZr}^{Ti}+{\tilde{D}}_{NbNb}^{Ti}>0,$$

(16) $${\tilde{D}}_{ZrZr}^{Ti}\times {\tilde{D}}_{NbNb}^{Ti}-{\tilde{D}}_{ZrNb}^{Ti}\times {\tilde{D}}_{NbZr}^{Ti}>0,$$

(17) $${\left({\tilde{D}}_{ZrZr}^{Ti}+{\tilde{D}}_{NbNb}^{Ti}\right)}^{2}-4\left({\tilde{D}}_{ZrZr}^{Ti}\times {\tilde{D}}_{NbNb}^{Ti}-{\tilde{D}}_{ZrNb}^{Ti}\times {\tilde{D}}_{NbZr}^{Ti}\right)\geq 0.$$

The impurity diffusion coefficients that were obtained by Hall methods are listed in Table 3, and also illustrated in Figure 4, along with the inter-diffusion coefficients. In Figure 4a,b, the main inter-diffusion coefficients coincide with the impurity diffusion coefficients when the concentration of diffusion elements tends to 0.

Scrutinizing the map of the diffusion coefficients in Figure 4, it can also be observed that, when the concentration of the gradient elements *j* tends to 0, the limit of main inter-diffusion coefficients ${\tilde{D}}_{ii}^{k}$ will match the binary diffusion coefficients from literature [33] well, and when the concentration of the diffusion elements *i* tend to 0, the limits of cross inter-diffusion coefficients ${\tilde{D}}_{ij}^{k}$ will be 0. The main diffusion coefficients are larger than the cross ones and they showed less fluctuation than cross ones. Values of ${\tilde{D}}_{MoZr}^{Ti}$ are negative, which means that the element Zr has a negative effect on the diffusion of Mo in Ti-based alloys.

**Figure 5:**
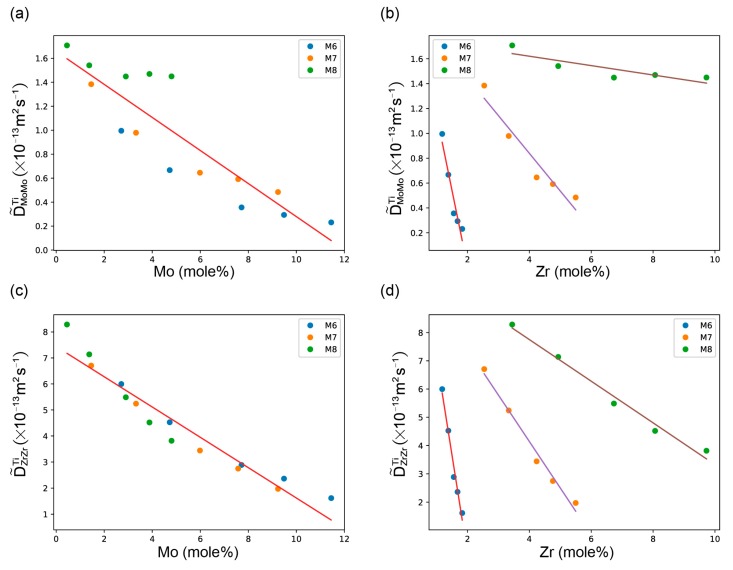
The variation of ternary inter-diffusion coefficients with the compositions: (**a**) ${\tilde{D}}_{MoMo}^{Ti}$ with Mo, (**b**) ${\tilde{D}}_{MoMo}^{Ti}$ with Zr, (**c**) ${\tilde{D}}_{ZrZr}^{Ti}$ with Mo and (**d**) ${\tilde{D}}_{ZrZr}^{Ti}$ with Zr at 1373 K.

The variation of ${\tilde{D}}_{MoMo}^{Ti}$ and ${\tilde{D}}_{ZrZr}^{Ti}$ with the composition of Mo and Zr at 1373 K are presented in Figure 5. It can be found that ${\tilde{D}}_{MoMo}^{Ti}$ decrease with the increase concentration of Mo in Figure 5a. In Figure 5b, ${\tilde{D}}_{MoMo}^{Ti}$ also decrease with the increase concentration of Zr. However, the decreasing rate varies with different diffusion Mo contents (the three colors correspond to inter-diffusion coefficients extracted from three different diffusion couples, M6, M7, and M8). In general, a great ratio of x(Zr)/x(Mo) slows down the process of decrease. As to ${\tilde{D}}_{ZrZr}^{Ti}$, as is shown in Figure 5c,d, show similar trends, like ${\tilde{D}}_{MoMo}^{Ti}$, when it varies with the concentration of Mo and Zr. From these phenomena, we can infer that the influence of element Mo on the variations of ${\tilde{D}}_{MoMo}^{Ti}$ and ${\tilde{D}}_{ZrZr}^{Ti}$ is much larger than Zr.

When extent to boundary, the impurity diffusion coefficients of Mo in Ti-Zr alloys, $D_{Mo\left(Ti-Zr\right)}^{*}$ show an increasing trend with the increase of Zr concentration. The impurity diffusion coefficients of Zr in Ti-Mo alloys $D_{Zr\left(Ti-Mo\right)}^{*}$ show a decreasing trend with the increase of Mo concentration.

**Figure 6:**
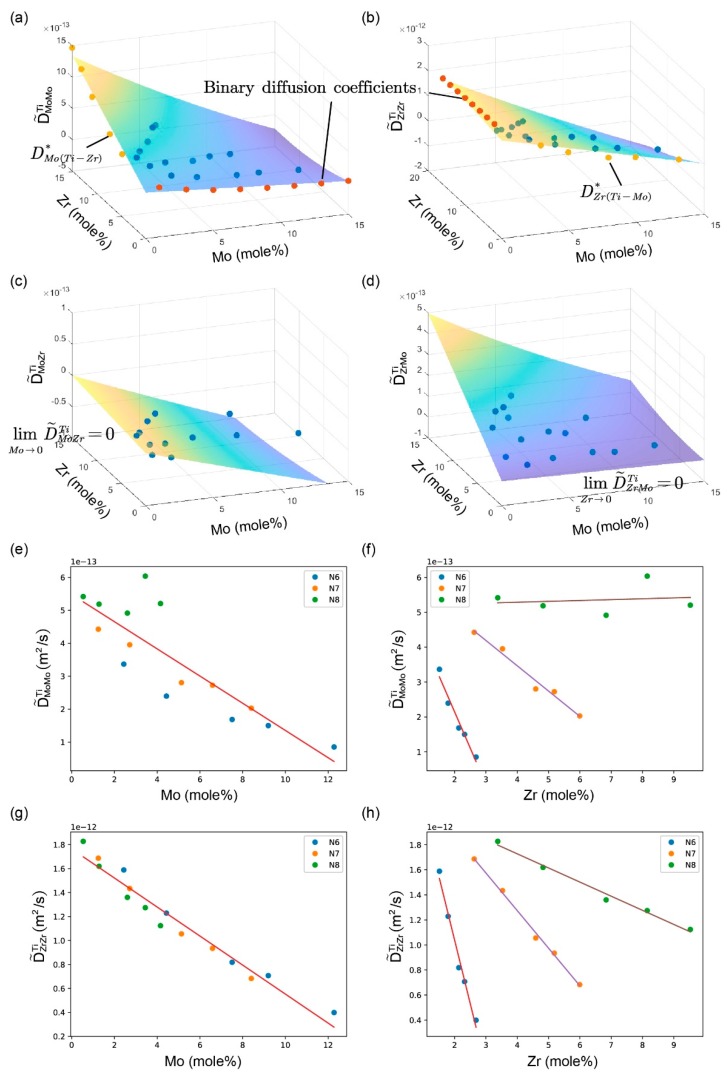
The 3D plot of the ternary inter-diffusion coefficients (**a**) ${\tilde{D}}_{MoMo}^{Ti}$, (**b**) ${\tilde{D}}_{ZrZr}^{Ti}$, (**c**) ${\tilde{D}}_{MoZr}^{Ti}$, and (**d**) ${\tilde{D}}_{ZrMo}^{Ti}$ in the bcc Ti-Mo-Zr alloys and the variation of ternary inter-diffusion coefficients with the compositions: (**e**) ${\tilde{D}}_{MoMo}^{Ti}$ with Mo, (**f**) ${\tilde{D}}_{MoMo}^{Ti}$ with Zr, (**g**) ${\tilde{D}}_{ZrZr}^{Ti}$ with Mo, and (**h**) ${\tilde{D}}_{ZrZr}^{Ti}$ with Zr at 1473 K.

Figure 6 presents the three-dimensional (3D) plot of diffusion coefficients in the bcc Ti-Mo-Zr alloys and the variations of ${\tilde{D}}_{MoMo}^{Ti}$ and ${\tilde{D}}_{ZrZr}^{Ti}$ with of the concentration of Mo and Zr at 1473 K. They showed similar trends as those at 1373 K.

### 4.2. Atomic Mobilities in bcc Ti-Zr-Mo System

The DICTRA module that was embedded in ThermoCalc software [21,22] was employed to obtain the atomic mobility parameters for the Ti-Mo-Zr ternary bcc alloys. Mobility parameters of boundary binary systems were taken from Liu’s work [33,34]. Thermodynamic description of boundary binary systems were also the same as Liu’s work [40,41,42]. Thermodynamic modeling of bcc phase in the ternary system can be carried out by simply extrapolation based on three binary systems. By assessing the experimental diffusion coefficients in this work, a set of self-consistent atomic mobility parameters were obtained, as listed in Table 4.

**Figure 7:**
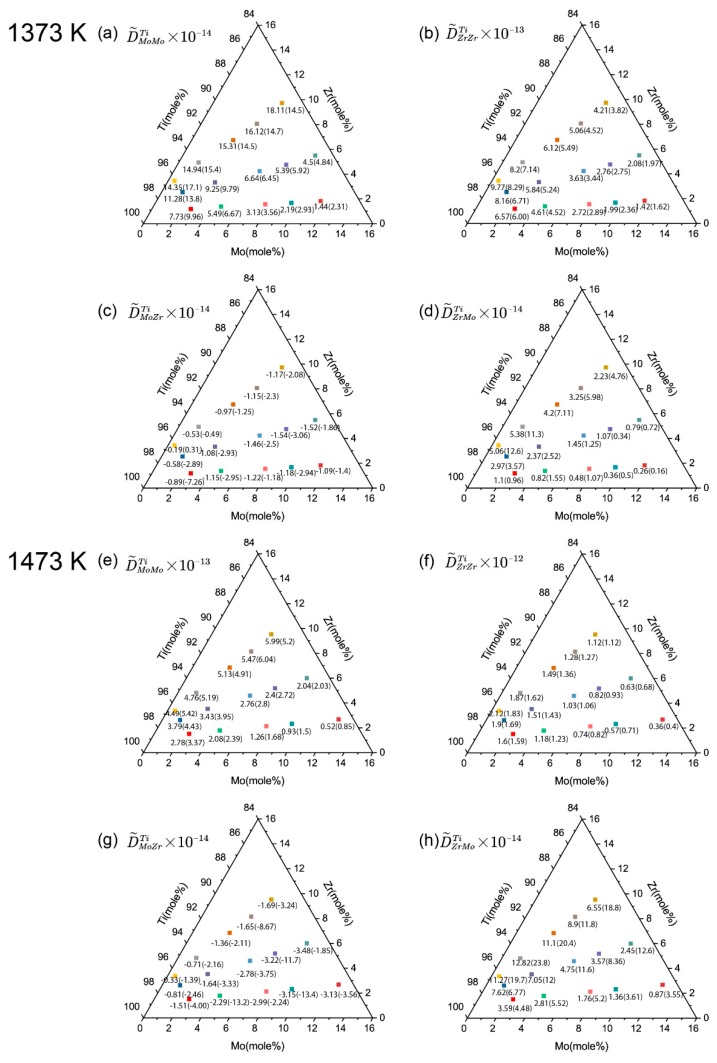
Calculated inter-diffusion coefficients of the ternary Ti-Mo-Zr system compared with the experimental measurements (in brackets) in this work: (**a**) ${\tilde{D}}_{MoMo}^{Ti}$, (**b**) ${\tilde{D}}_{ZrZr}^{Ti}$, (**c**) ${\tilde{D}}_{MoZr}^{Ti}$ and (**d**) ${\tilde{D}}_{ZrMo}^{Ti}$ at 1373 K and (**e**) ${\tilde{D}}_{MoMo}^{Ti}$, (**f**) ${\tilde{D}}_{ZrZr}^{Ti}$, (**g**) ${\tilde{D}}_{MoZr}^{Ti}$ and (**h**) ${\tilde{D}}_{ZrMo}^{Ti}$ at 1473 K.

Using these parameters and DICTRA software, the inter-diffusion coefficients at the intersection composition at 1373 K and 1473 K were calculated and compared with the experimental results, as shown in Figure 7. The calculated results agree with the main inter-diffusion coefficients well, within the error allowed. In contrast, the cross coefficients show certain fluctuation with a large error range. Since the calculated and experimental cross coefficients show values in the same order of magnitude, and a same variation tendency with compositions, the accessed mobilities are feasible to represent the cross coefficients.

**Figure 8:**
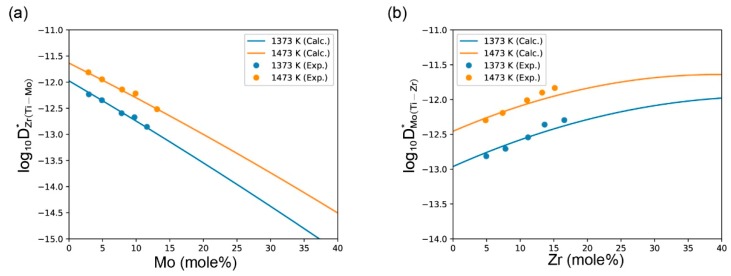
Calculated impurity diffusion coefficients (**a**) $D_{Zr\left(Ti-Mo\right)}^{*}$ and (**b**) $D_{Mo\left(Ti-Zr\right)}^{*}$ as compared with the experimental data.

Figure 8 shows the comparison of the calculated impurity diffusion coefficients and the experimental results. The calculated impurity diffusion coefficients also agree well with the experimental results.

**Figure 9:**
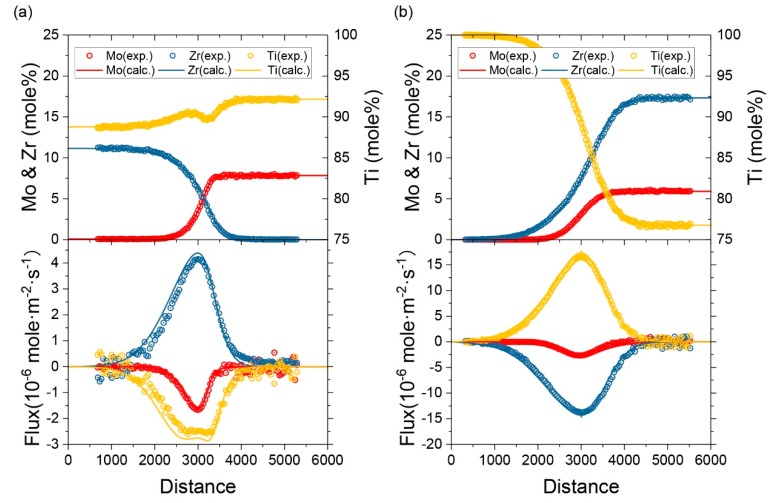
Predicted composition-distance profiles and inter-diffusion fluxes of M3 and N8 as compared with experimental data.

**Figure 10:**
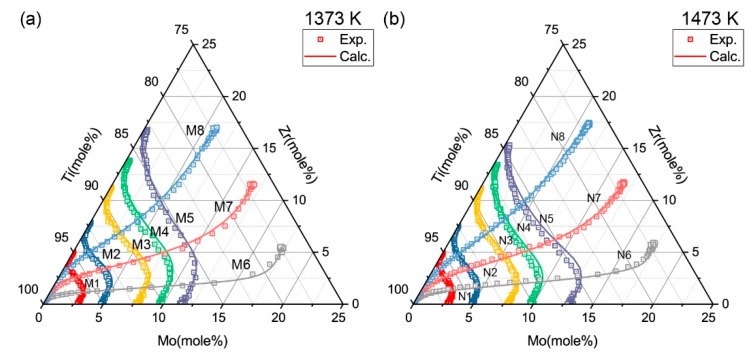
Simulated diffusion paths for diffusion couples compared with the experimental measurements: (**a**) 1373 K and (**b**) 1473 K.

In order to further verify the atomic mobility parameters that were obtained in the present work, the simulation of several diffusion couples were implemented. The diffusion simulation was set up to model the semi-infinite ternary diffusion couples in experiment. It was conducted using the same initial conditions and heat treatment processes in the experiment. The predict composition-distance profiles show good agreement with the experimental profiles. For instance, the predicted composition-distance profiles and fluxes of couple M3 and N8 compared with experimental data are exhibited in Figure 9 and Figure 10 presented the simulated diffusion paths of 16 diffusion couples annealed at 1373 K and 1473 K. The calculated results show good agreement with the experimental results.

## 5. Conclusions

In this work, two set of diffusion couples of bcc Ti-Mo-Zr alloys were made and annealed at 1373 K for 72 h and 1473 K for 48 h, respectively. The composition-distance profiles of these diffusion couples were determined using EPMA. The ternary inter-diffusion coefficients and impurity diffusion coefficients were extracted using the Whittle-Green and Hall method. Based on the experimental results and thermodynamic descriptions, as well as atomic mobility parameters of the sub-binary systems of Ti-Mo-Zr system, an atomic mobility database for the bcc phase in the Ti-Mo-Zr ternary system was developed. Inter-diffusion coefficients at the intersection points of diffusion couples and impurity diffusion coefficients were calculated using the database and compared with the data extracted directly from the composition-distance profiles. In addition, simulations of the diffusion couples with the same initial conditions and heat treatment processes of the experiment were proceeded. The composition-distance profiles and diffusion paths were compared with the experimental results. All of the calculated results show good agreement with experimental data.

## Figures and Tables

**Table 1:** Compositions of diffusion couples.

Temperature (K)	Diffusion Couples	Composition (mole %)
1373	M1	Ti-2.95Mo/Ti-4.95Zr
	M2	Ti-4.90Mo/Ti-7.81Zr
	M3	Ti-7.83Mo/Ti-11.17Zr
	M4	Ti-9.74Mo/Ti-13.64Zr
	M5	Ti-11.61Mo/Ti-16.57Zr
	M6	Pure Ti/Ti-17.18Mo-5.48Zr
	M7	Pure Ti/Ti-11.63Mo-11.51Zr
	M8	Pure Ti/Ti-5.91Mo-16.90Zr
1473	N1	Ti-2.85Mo/Ti-4.85Zr
	N2	Ti-4.92Mo/Ti-7.39Zr
	N3	Ti-7.90Mo/Ti-11.02Zr
	N4	Ti-9.90Mo/Ti-13.27Zr
	N5	Ti-13.11Mo/Ti-15.15Zr
	N6	Pure Ti/Ti-17.14Mo-5.81Zr
	N7	Pure Ti/Ti-11.67Mo-11.61Zr
	N8	Pure Ti/Ti-5.91Mo-17.32Zr

**Table 2:** Inter-diffusion coefficients in the bcc Ti-Mo-Zr alloys at 1373 K and 1473 K.

Temp. (K)	Diffusion Couple	Intersection Composition (mole %)		Inter-Diffusion Coefficients (m^2^·s^−1^)			
		Mo	Zr	${\tilde{D}}_{MoMo}^{Ti}$	${\tilde{D}}_{MoZr}^{Ti}$	${\tilde{D}}_{ZrMo}^{Ti}$	${\tilde{D}}_{ZrZr}^{Ti}$
				×10^−14^	×10^−14^	×10^−14^	×10^−13^
1373	M1-M6	2.71	1.18	9.96 ± 1.12	−7.26 ± 5.24	0.96 ± 0.38	6.00 ± 0.42
	M1-M7	1.46	2.54	13.84 ± 0.56	−2.89 ± 1.72	3.57 ± 2.24	6.71 ± 0.37
	M1-M8	0.45	3.45	17.08 ± 1.81	0.31 ± 0.25	12.63 ± 5.54	8.29 ± 0.31
	M2-M6	4.73	1.38	6.67 ± 0.60	−2.95 ± 1.93	1.55 ± 0.20	4.52 ± 0.11
	M2-M7	3.32	3.33	9.79 ± 0.25	−2.93 ± 0.27	2.52 ± 1.47	5.24 ± 0.10
	M2-M8	1.38	4.93	15.41 ± 0.56	−0.49 ± 0.33	11.30 ± 1.09	7.14 ± 0.10
	M3-M6	7.72	1.56	3.56 ± 0.41	−1.18 ± 0.41	1.07 ± 0.13	2.89 ± 0.10
	M3-M7	5.99	4.24	6.45 ± 0.11	−2.50 ± 0.36	1.25 ± 0.60	3.44 ± 0.15
	M3-M8	2.90	6.74	14.48 ± 0.28	−1.25 ± 0.48	7.11 ± 0.99	5.49 ± 0.15
	M4-M6	9.49	1.68	2.93 ± 0.17	−2.94 ± 1.04	0.50 ± 0.15	2.36 ± 0.06
	M4-M7	7.58	4.76	5.92 ± 0.28	−3.06 ± 0.58	0.34 ± 0.71	2.75 ± 0.14
	M4-M8	3.88	8.07	14.69 ± 0.93	−2.30 ± 0.84	5.98 ± 1.07	4.52 ± 0.04
	M5-M6	11.45	1.83	2.31 ± 0.29	−1.40 ± 1.31	0.16 ± 0.46	1.62 ± 0.06
	M5-M7	9.24	5.50	4.84 ± 0.60	−1.86 ± 0.68	0.72 ± 0.78	1.97 ± 0.02
	M5-M8	4.80	9.73	14.49 ± 1.04	−2.08 ± 0.50	4.76 ± 3.54	3.82 ± 0.16
				×10^−13^	×10^−14^	×10^−13^	×10^−12^
1473	N1-N6	1.524	3.605	3.37 ± 0.18	−4.00 ± 2.19	0.45 ± 0.22	1.59 ± 0.06
	N1-N7	2.597	1.898	4.43 ± 0.01	−2.46 ± 1.08	0.68 ± 0.40	1.69 ± 0.02
	N1-N8	3.365	0.81	5.42 ± 0.13	−1.39 ± 1.30	1.97 ± 1.16	1.83 ± 0.05
	N2-N6	1.707	4.399	2.39 ± 0.19	−13.24 ± 4.86	0.55 ± 0.06	1.23 ± 0.01
	N2-N7	3.589	3.386	3.95 ± 0.12	−3.33 ± 2.17	1.20 ± 0.23	1.43 ± 0.03
	N2-N8	5.701	1.951	5.19 ± 0.12	−2.16 ± 1.40	2.38 ± 0.21	1.62 ± 0.02
	N3-N6	2.43	8.144	1.68 ± 0.04	−2.24 ± 2.12	0.52 ± 0.09	0.82 ± 0.02
	N3-N7	4.724	5.243	2.80 ± 0.05	−3.75 ± 0.51	1.16 ± 0.15	1.06 ± 0.02
	N3-N8	6.881	2.508	4.91 ± 0.08	−2.11 ± 0.76	2.04 ± 0.51	1.36 ± 0.03
	N4-N6	2.552	8.725	1.50 ± 0.09	−13.40 ± 4.90	0.36 ± 0.10	0.71 ± 0.01
	N4-N7	5.489	6.461	2.72 ± 0.17	−11.73 ± 2.86	0.84 ± 0.06	0.93 ± 0.01
	N4-N8	8.913	3.466	6.04 ± 0.46	−8.67 ± 2.82	1.18 ± 0.19	1.27 ± 0.02
	N5-N6	3.438	12.795	0.85 ± 0.03	−3.56 ± 1.06	0.36 ± 0.06	0.40 ± 0.01
	N5-N7	6.723	8.091	2.03 ± 0.07	−1.85 ± 0.97	1.26 ± 0.26	0.68 ± 0.01
	N5-N8	9.993	3.932	5.20 ± 0.44	−3.24 ± 2.66	1.88 ± 0.66	1.12 ± 0.03

**Table 3:** Impurity diffusion coefficients of Zr in Ti-Mo and Mo in Ti-Zr alloys at 1373 K and 1473 K.

Temperature/K	Composition	Impurity Diffusion Coefficients (×10^−13^ m^2^·s^−1^)	Composition	Impurity Diffusion Coefficients (×10^−13^ m^2^·s^−1^)
1373	$D_{Zr\left(Ti-2.95Mo\right)}^{*}$	5.85 ± 0.58	$D_{Mo\left(Ti-4.95Zr\right)}^{*}$	1.54 ± 0.09
	$D_{Zr\left(Ti-4.90Mo\right)}^{*}$	4.51 ± 0.88	$D_{Mo\left(Ti-7.81Zr\right)}^{*}$	1.96 ± 0.62
	$D_{Zr\left(Ti-7.83Mo\right)}^{*}$	2.55 ± 0.05	$D_{Mo\left(Ti-11.17Zr\right)}^{*}$	2.86 ± 0.71
	$D_{Zr\left(Ti-9.74Mo\right)}^{*}$	2.13 ± 0.14	$D_{Mo\left(Ti-13.64Zr\right)}^{*}$	4.37 ± 1.68
	$D_{Zr\left(Ti-11.61Mo\right)}^{*}$	1.39 ± 0.15	$D_{Mo\left(Ti-16.57Zr\right)}^{*}$	5.07 ± 1.45
1473	$D_{Zr\left(Ti-2.89Mo\right)}^{*}$	15.45 ± 1.22	$D_{Mo\left(Ti-4.85Zr\right)}^{*}$	5.04 ± 1.24
	$D_{Zr\left(Ti-4.92Mo\right)}^{*}$	11.31 ± 0.74	$D_{Mo\left(Ti-7.39Zr\right)}^{*}$	6.42 ± 2.38
	$D_{Zr\left(Ti-7.90Mo\right)}^{*}$	7.24 ± 0.07	$D_{Mo\left(Ti-11.02Zr\right)}^{*}$	9.78 ± 1.82
	$D_{Zr\left(Ti-9.90Mo\right)}^{*}$	6.08 ± 0.26	$D_{Mo\left(Ti-13.27Zr\right)}^{*}$	12.61 ± 3.46
	$D_{Zr\left(Ti-13.11Mo\right)}^{*}$	3.04 ± 0.17	$D_{Mo\left(Ti-15.15Zr\right)}^{*}$	14.66 ± 2.39

**Table 4:** Atomic mobility parameters for the bcc phase of the Ti-Mo-Zr ternary system.

Mobility	Parameter, J/mole	Reference
Mobility of Mo		
$Q_{Mo}^{Mo}$	−479740.87 − 63.98·T	[33]
$Q_{Mo}^{Ti}$	−196255.40 − 105.21·T	[33]
$Q_{Mo}^{Zr}$	−154895.63 − 140.91·T (T ≤ 1450 K) −214913.77 − 114.17·T (T ≥ 1450 K)	[34]
${}^{0}Q_{Mo}^{Mo,Ti}$	−24153.22 − 45.32·T	[33]
${}^{1}Q_{Mo}^{Mo,Ti}$	−61804.04	[33]
$Q_{Mo}^{Mo,Zr}$	150325.48 + 10.03·T	[34]
$Q_{Mo}^{Ti,Zr}$	−268357.13 + 283.18·T	This work
${}^{0}Q_{Mo}^{Mo,Ti,Zr}$	53485.75	This work
${}^{1}Q_{Mo}^{Mo,Ti,Zr}$	2707991.87	This work
${}^{2}Q_{Mo}^{Mo,Ti,Zr}$	−719018.41	This work
Mobility of Ti		
$Q_{Ti}^{Mo}$	−435701.23 − 72.67·T	[33]
$Q_{Ti}^{Ti}$	−151989.95 − 127.37·T	[33]
$Q_{Ti}^{Zr}$	−140356.54 − 138.12·T	[33]
${}^{0}Q_{Ti}^{Mo,Ti}$	−91728.48 + 64.56·T	[33]
${}^{1}Q_{Ti}^{Mo,Ti}$	−96300.05	[33]
${}^{0}Q_{Ti}^{Ti,Zr}$	−15826.04 + 62.55·T	[33]
${}^{1}Q_{Ti}^{Ti,Zr}$	8243.54	[33]
${}^{0}Q_{Ti}^{Mo,Ti,Zr}$	−1304851.67	This work
${}^{1}Q_{Ti}^{Mo,Ti,Zr}$	−6019149.28	This work
${}^{2}Q_{Ti}^{Mo,Ti,Zr}$	−1128202.89	This work
Mobility of Zr		
$Q_{Zr}^{Mo}$	−464587.32 − 64.72·T	[34]
$Q_{Zr}^{Ti}$	−131670.56 − 133.36·T	[33]
$Q_{Zr}^{Zr}$	−104624.81 − 163.15·T (T≤1573 K) −161543.53 − 126.10·T (T≥1573 K)	[33]
$Q_{Zr}^{Mo,Ti}$	−81189.65 + 88.51·T	This work
$Q_{Zr}^{Mo,Zr}$	210325.67 + 15.19·T	[34]
${}^{0}Q_{Zr}^{Ti,Zr}$	−12581.03 + 33.38·T	[33]
${}^{1}Q_{Zr}^{Ti,Zr}$	2898.60	[33]
${}^{0}Q_{Zr}^{Mo,Ti,Zr}$	2864755.37	This work
${}^{1}Q_{Zr}^{Mo,Ti,Zr}$	−340701.76	This work
${}^{2}Q_{Zr}^{Mo,Ti,Zr}$	−609499.74	This work
